# Dynamics-based transition states reveal solvent cage effect and S_N_2 transition state motion in Lewis acid catalyzed stereoselective tertiary alcohol nucleophilic substitution reactions

**DOI:** 10.1039/d5sc05616k

**Published:** 2025-10-16

**Authors:** Anthony J. Schaefer, Trevor Mallavia, Daniel H. Ess

**Affiliations:** a Department of Chemistry and Biochemistry, Brigham Young University Provo Utah 84604 USA dhe@byu.edu

## Abstract

It has been known for a very long time that optically active tertiary sp^3^ carbons with a leaving group can undergo substitution with some selectivity for configuration inversion. The Weinstein model proposes that this stereoselectivity is governed by a S_N_1 mechanism involving an intimate ion pair, but transition states for this type of reaction pathway have not yet been examined with explicit solvent. Here we have used quantum mechanics/molecular mechanics (QM/MM) based direct dynamics trajectories in explicit solvent to identify and characterize the S_N_1 reaction mechanism for a model tertiary alcohol conversion to an isonitrile with Sc(OTf)_3_ in TMSCN solvent that was experimentally reported have high configuration inversion. Using dynamics trajectories, we located both the leaving group loss and nucleophilic attack transition states along with the intimate ion pair intermediate. The stereo-determining nucleophilic attack transition state from the intimate ion pair has significant S_N_2 character with dynamically coupled nitrogen–carbon bond formation and leaving group ejection. Comparison with continuum solvent trajectories revealed that the intimate ion pair intermediate is the result of the solvent cage and that without the cage a free carbocation intermediate would be formed leading either little or no stereoselectivity.

## Abstract 2

The stereo-determining nucleophilic attack transition state from the intimate ion pair has significant S_N_2 character with dynamically coupled nitrogen–carbon bond formation and leaving group ejection.

## Graphical abstract

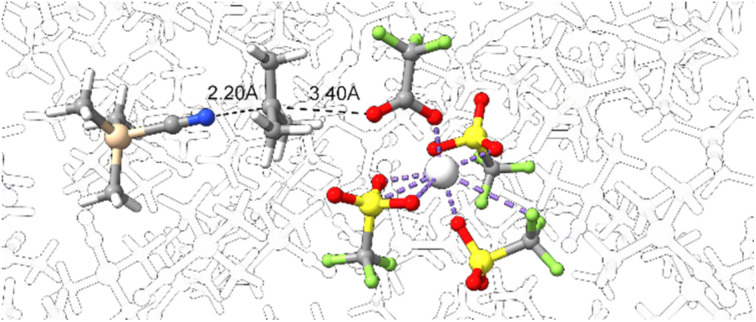

## Introduction

**Fig. 1:**
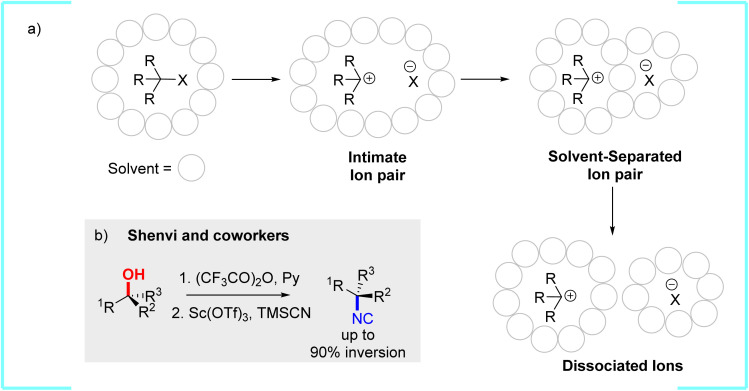
(a) Outline of the proposed intermediates that can occur during a sp^3^ nucleophilic substitution involving a carbocation intermediate. (b) Overview of tertiary alcohol substitution reported by Shenvi and coworkers.^22^

There is a long history of experimental kinetic, stereochemical, salt effect, isotope labeling, and solvent mechanistic studies probing the nature of intermediates during nucleophilic substitution reactions of secondary and tertiary sp^3^ carbon centers.^1^ Classic studies by Winstein,^2–4^ Levy,^5^ and others have led to an established mechanistic framework with the involvement of ion pairs and solvent effects (Fig. 1a). The first step (often called stage) involves carbon-leaving group bond heterolysis to generate an intimate ion pair,^6,7^ which can also be referred to as a solvent caged ion pair.^8^ The second step involves the evolution of the intimate ion pair into a solvent separated ion pair where there is at least one solvent molecule separating the ions.^9,10^ It is often unclear in many cases the lifetime of the solvent-separated ion pair and if or for how long it retains any memory of the intimate ion pair state. Last, this solvent-separated ion pair evolves into fully separated ions with a carbocation intermediate surrounded only by solvent, presumably with no short-range or long-range interaction with the leaving group. The separated ions are also assumed to be fully equilibrated with solvent and have no memory of each other.

It has been known for a very long time that for secondary and tertiary sp^3^ carbon substitution reactions with optically active chiral starting compounds complete racemization is uncommon,^11^ which is the expected outcome if the nucleophile or solvent reacts with the carbocation at the dissociated ions. In reactions where stereochemistry has been tracked the percentage of inversion selectivity generally ranges from 5% to 60%.^9^ However, for alkyl chlorides there are a few select examples that show inversion can reach >90% and this high of inversion depends on the exact alkyl halide, solvent to nucleophile ratio, and temperature.^12^ For tertiary alcohols and esters the relative amount of inversion is often low and highly sensitive to the starting compound,^13^ although there are now a few very recent studies that do show high selectivity.^14–21^ Specifically, it was remarkable when Shenvi and coworkers reported a protocol for conversion of optically active chiral alcohols to their corresponding isonitriles (and ultimately to tertiary alkyl amine) with high configuration inversion often around 90% (Fig. 1b).^22,23^ The alcohol group was first converted to the trifluoroacetate ester and then displaced using a high concentrations of trimethylsilyl cyanide (TMSCN) with Sc(OTf)_3_ as the Lewis acid catalyst. Control experiments by Shenvi suggest that TMSCN is unlikely to be a Lewis acid in bond heterolysis and it only delivers the nucleophile.^22^ Also, the addition of hindered Brønsted bases (*e.g.* 2,6-di-*t*-butylpyridine) did not inhibit the reaction, excluding Brønsted acid catalysis.

For tertiary alkyl chloride or alcohol carbon substitution reactions where racemization was incomplete the prevailing rationale within the Weinstein mechanistic model is that at the intimate ion pair intermediate the dislodged leaving group blocks one side of the carbocation intermediate. Similarly, for the alcohol substitution reaction reported by Shenvi it was proposed that the ratio of inversion to retention is controlled by the rate of TMSCN capture of the intimate ion pair *versus* the rate of formation of the solvent separated ion pair.^22^ Surprisingly, to date, using quantum-chemical calculations there has been no comprehensive evaluation of sp^3^ carbon substitution reactions that detail the origin of significant selectivity for inversion. Therefore, it remains unknown the exact intermediate or stage necessary for nucleophilic attack and leaving group loss to occur to yield high inversion selectivity. Also, it is unclear the role of dynamic effects in coupling the motion of nucleophilic bonding with carbon center and leaving group loss along with the reorganization of solvent.

Based on these unresolved questions surrounding tertiary sp^3^ alcohol substitution reactions we decided to use density functional theory (DFT) to calculate the Sc^III^ catalyzed alcohol to isonitrile conversion using a model *tert*-butyl substrate. Modeling this type of reaction with traditional DFT based potential energy surface calculations is complicated because there is likely the need to have sufficient explicit solvation^24^ and location of transition states and intermediates are difficult because surfaces involving solvated ion pairs are often very flat.^25,26^ Therefore, to model this reaction we used quasiclassical direct dynamics trajectories with a sphere of explicit solvent (treated classically) to locate dividing surface structures that represent the kinetic bottleneck for reaction steps.^27^ This analysis led to a picture of the alcohol substitution reaction where there is an initial bond heterolysis transition state, and the leaving group dynamic motion is slow resulting in formation of an intimate/tight ion pair with a lifetime greater than several picoseconds (ps). Following this intermediate for several ps showed that it retained the tight ion pair configuration without solvent dividing the ions. Comparison to continuum solvent dynamics trajectories revealed that without the explicit solvent cage the leaving group is completely ejected and there is formation of separated ions. After the intimate ion pair is formed in solvent there is a second transition state for nucleophilic attack of the carbocation center and this attack is dynamically coupled with full leaving group ejection revealing that the transition state has significant S_N_2 character/motion, which is different than simple steric facial blocking, and this provides a rationale for the observed stereoselectivity.

## Methods

All DFT and ONIOM calculations were performed using the Gaussian 16 software.^28^ DFT calculations, including the DFT ONIOM layer, used the M06-2X/6-31G**[LANL2DZ for Sc] method and basis set.^29^ For implicit solvent trajectories the PCM^30,31^ model with acetonitrile parameters were used. For explicitly solvated calculations, Packmol^32^ was used to generate initial configurations followed by equilibration (see below). The TMSCN solvent molecules, except for the nucleophile that was treated with DFT, were modeled using the UFF potential. While the Sc metal center is almost certainly solvated and has coordination with multiple TMSCN we kept the Sc(OTf)_3_ with *κ*^2^-coordination as a model to reduce the size of the system required to be modeled with DFT direct dynamics simulations. While this approximation certainly impacts the exact charge on the Sc center it likely does not change the qualitative conclusions about the transition states located. Indeed, we also modeled the key nucleophilic attack transition state with [Sc(TMSCN)_5_]^3+^ instead of Sc(OTf)_3_, and found a qualitatively similar outcome with dynamics trajectories. For the alcohol substrate, we used a model *tert*-butyl alcohol, which represents a truncated tertiary alcohol that Shenvi examined.

Direct molecular dynamics trajectories were carried out using our Milo^33^ software interfaced with Gaussian for computing energies and atomic forces. For explicitly solvated trajectories, ^*t*^Bu-OTFA (OTFA = trifluoroacetate), Sc(OTf)_3_, and one proximal TMSCN molecule were in the high (DFT) ONIOM layer with 150 TMSCN molecules added in the low (UFF) layer. Molecules in these trajectories were contained by a 20 Å radius sphere. Prior to NVE trajectories, the solvent was equilibrated at 298.15 K using the canonical sampling through velocity rescaling thermostat^34^ (CSVR) for at least 16.5 picoseconds (ps). Trajectories used quasiclassical normal mode sampling for the solute and classical sampling from the equilibration step for the solvent.

For trajectories with only *tert*-butyl carbocation and solvent, 150 TMSCN molecules packed around the *tert*-butyl cation in a 20 Å radius sphere. The system was equilibrated at 298.15 K. After equilibration, nine of the TMSCN molecules closest to the carbocation were selectively added to the DFT ONIOM layer that included the carbocation. The remainder of the solvent was treated with UFF.

Umbrella sampling was used to estimate the barrier for *tert*-butyl cation forming a bond with a TMSCN molecule. A CSVR thermostat was employed during both equilibration and production trajectories. The *tert*-butyl cation and one TMSCN molecule were in the DFT ONIOM layer and 100 TMSCN molecules were in the UFF ONIOM layer. These simulations were bounded by a 17 Å radius sphere. A force constant of 825 kcal mol^−1^ was applied to restrain the distance between the *tert*-butyl cation's central carbon and the TMSCN's nitrogen.

## Results and discussion

As discussed in the Introduction, substitution reactions at a tertiary sp^3^ carbon center that result in stereoselective configuration inversion are generally proposed to proceed through a two-step S_N_1 mechanism with an intimate ion pair intermediate (Fig. 1a),^11,14^ and the dislodged leaving group sterically blocks one side of the carbocation intermediate. A S_N_1 mechanism rather than a S_N_2 mechanism is generally proposed because there is the expectation that a S_N_2 mechanism would give complete stereoinversion since the incoming nucleophile motion is dynamically coupled with leaving group ejection. While not extensively discussed, there is also an underlying assumption that partial stereoselective configuration inversion does not result from competitive S_N_1 and S_N_2 mechanisms.^11^ This is because for a tertiary carbon center with a leaving group, a single-step S_N_2 transition state is assumed to be very high in energy and not competitive with carbon-leaving group bond heterolysis, and many quantum-chemical studies over the past few decades are consistent with this assumption.^35,36^ This can be rationalized by large steric repulsion between the incoming nucleophile with the groups attached to the sp^3^ carbon center,^37^ and this repulsion is apparently not compensated for by the new nucleophile-to-carbon bonding interaction until after complete carbon-leaving group bond heterolysis. Related, it is also well established based on quantum-chemical calculations that a single step frontside S_N_2 transition state is also very high in energy would not be competitive with a S_N_2 pathway in these tertiary substitution reactions.^36,37^

Based on the general S_N_1 reaction steps outlined in Fig. 1a Shenvi proposed that the high stereoinversion selectivity for conversion of optically active chiral tertiary alcohols to their corresponding isonitriles is determined by the rate of TMSCN capture of the intimate ion pair *versus* the rate of formation of the solvent separated ion pair.^22^ Therefore, our general goal was to evaluate the S_N_1 transition states for this reaction as well as characterize the key intimate ion pair intermediate. To date, there has been no comprehensive computational study that has identified the full reaction pathway for substitution reactions at a tertiary sp^3^ carbon center that result in stereoselective configuration inversion. It is therefore unknown how much the solvent impacts the formation and structure of the intimate ion pair and what role atomic motion plays in coupling nucleophilic bonding with the carbon center and leaving group loss.

While potential energy surface calculated S_N_2 transition states are often reported in the literature with varying levels of solvent treatment,^38–45^ there are only a few reports of S_N_1 transition-state structures such as Yamabe's analysis of benzyl chloride hydrolysis.^46^ In many cases an S_N_2 transition state located is described to have S_N_1 character. For example, Fiorot and coworkers recently examined explicit solvent effects and transition states for substitution reactions of isopropyl chloride,^47^ and only located S_N_2 transition states. For glycosylation reactions, Bernasconi and Liu used metadynamics simulations in explicit solvent to locate transition states and *ab initio* molecular dynamics simulations to characterize the timing of nucleophile attack and leaving group exit.^48^ Depending on the nucleophile and solvent, Bernasconi and Liu found that the glycosylation reaction could follow a S_N_1 pathway with an oxocarbenium intermediate generated for several ps.

The S_N_1 transition-state structures reported in the literature are generally only for the first reaction step involving carbon-leaving group bond heterolysis and not for subsequent nucleophilic attack, if the nucleophile and leaving group are different. The reason for the lack of S_N_1 transition states is that the potential energy surface is very flat in the vicinity of the intimate ion pair intermediate and solvent effects likely play an important role in the structure and motion. This prompted us to examine the intimate ion pair intermediate and identify transition states in explicit solvent using molecular dynamics simulations. Briefly, because the S_N_1 process has two main reaction coordinate components, nucleophile-carbon distance and carbon-leaving group distance we generated a grid of structures that vary these two distances. Each of these structures with fixed distances was then surrounded by a sphere of 160 TMSCN solvents. The solvent was then equilibrated for 10 ps using a thermostat. Following equilibration reactive direct dynamics trajectories were then initiated and propagated for up to 2 ps. The first S_N_1 transition state was identified by the structure that gave closest to an equal ratio of forming reactants and forming the intimate ion pair, which provides an approximate dividing surface. The second S_N_1 transition was identified by the structure that gave the closest to an equal ratio of forming the intimate ion pair and forming the adduct with the TMSCN nucleophile.

**Fig. 2:**
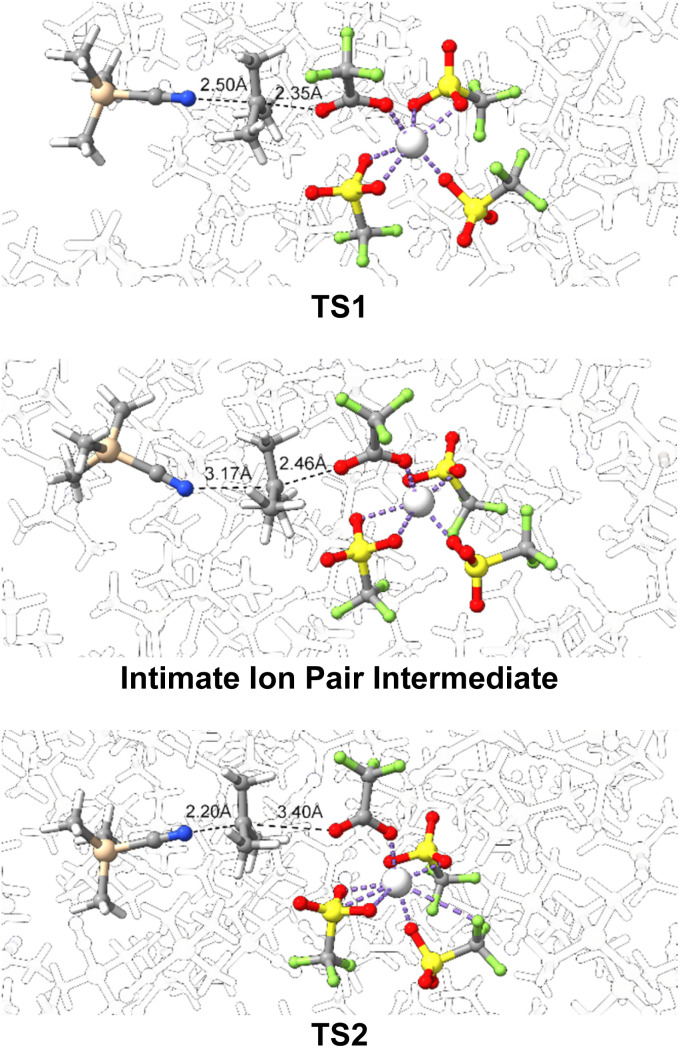
Top: substitution transition state for C-OTFA bond heterolysis. Middle: intimate ion pair intermediate. Bottom: substitution transition state for nucleophile capture of the tertiary carbon center. TMSCN solvents are transparent or hidden for visual clarity.

Fig. 2 shows the structures for the substitution reaction pathway identified by using direct dynamics simulations in explicit TMSCN solvent for the reaction between TMSCN and ^*t*^Bu-OTFA (OTFA = trifluoroacetate) with Sc(OTf)_3_. In the first transition state, TS1, the ^*t*^Bu-OTFA bond is stretched to 2.35 Å and the carbon center of the *tert*-butyl fragment is planar. While the TMSCN-carbon bond is not highly developed in TS1 there is some engagement with an interaction distance of 2.50 Å. With this primed engagement of the nucleophile one possible explanation for the selectivity for stereoinversion is that decent from TS1 would result in some trajectories skipping the intimate ion pair and directly resulting in forming the TMSCN-carbon bond, which would be a dynamically one step reaction. However, all the molecular dynamics trajectories started at TS1 moving in the forward direction toward the intimate ion pair only showed elongation of the carbon–oxygen distance to about 2.46 Å and no formation of the nitrogen–carbon bond. In fact, there is slight disengagement of the TMSCN nucleophile from the carbon and the nitrogen–carbon distance elongates to about 3.17 Å. The trajectories indicate that the intimate ion pair is a genuine intermediate and has a lifetime of at least 2 ps.

The second transition state, TS2, involves formation of the nitrogen–carbon bond at 2.20 Å. This transition state features a carbon-OTFA distance that at 3.40 Å is highly elongated compared to the intimate ion pair intermediate. This implies that TS2 has motion akin to a S_N_2 type transition state where there is concerted and dynamically coupled motion for formation of the nitrogen–carbon bond with leaving group ejection. This means while the dislodged leaving group does sterically block one side of the carbocation intermediate, the origin of selectivity is also due to a second S_N_1 transition state with S_N_2 type motion. Therefore, in this selectivity model, which is consistent with Shenvi's original proposal and Winstein's general ideas, there is competition between TS2 and dissociation to solvent separated ions. However, it is possible that there could be a concerted transition state for TMSCN to displace the OTFA/Sc group from the frontside before a solvent separated ion pair is fully formed. We did not model this transition state since it would lead to the minor product.

**Fig. 3:**
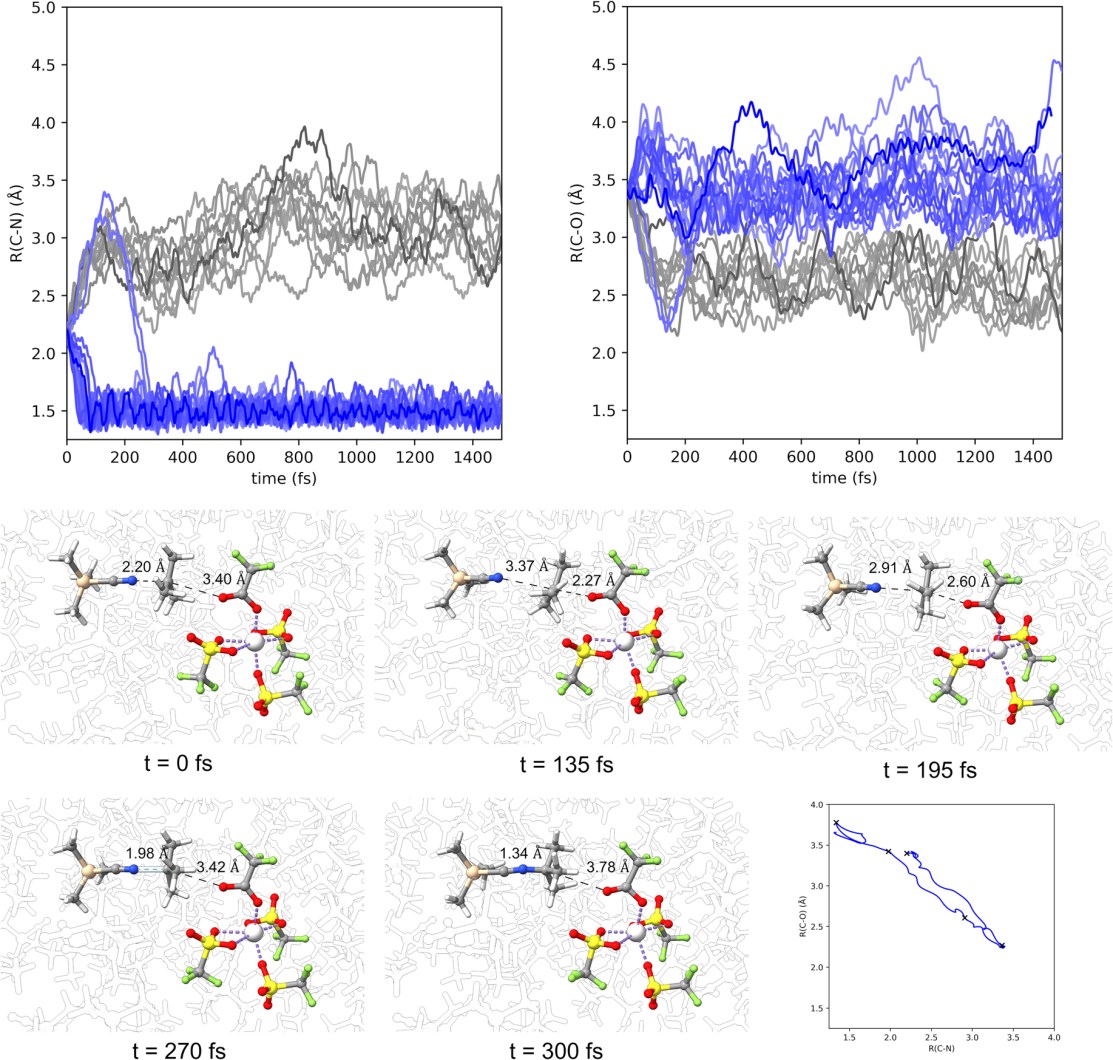
Top: plots of TMSCN-carbon (labeled as C–N) distance and carbon-OTFA distance *versus* time (femtoseconds (fs)) for representative trajectories starting at TS2. Blue colored lines represent the distances moving in the forward direction towards the intimate ion pair. The grey colored lines represent the distances moving in the reverse direction toward the reactants. Bottom: snapshots of a representative trajectory forming the nitrogen–carbon bond starting from TS2. TMSCN solvents are transparent or hidden for visual clarity.

To examine this potentially correlated transition state motion, Fig. 3 plots the forming nitrogen–carbon bond distance and the elongating C-OTFA distance for several representative trajectories starting at TS2 and moving in the forward direction towards the substitution product. These plots show that the nitrogen–carbon bond is generally formed very fast and within 50 fs. These plots also show that the C-OTFA distance increase indeed occurs simultaneously to the nitrogen–carbon bond formation. Snapshots of a slower to evolve trajectory are shown at the bottom of Fig. 3. In this example, the carbocation initially slides back towards forming the reactants then reverses course and forms the C–N bond, with the C–O distance increasing as the C–N distance decreases, demonstrating dynamically coupled motion in a S_N_2 type transition state.

**Fig. 4:**
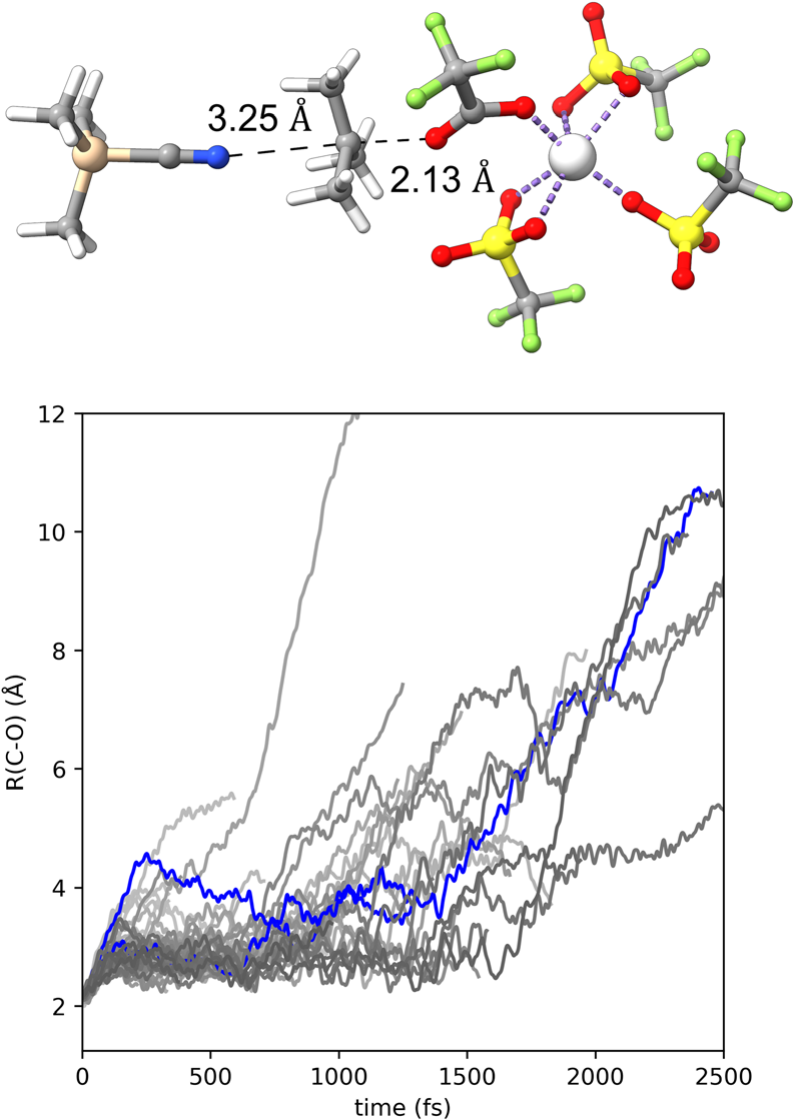
Top: continuum solvent optimized transition-state structure involving carbon–oxygen bond heterolysis. Bottom: plot of carbon–oxygen bond distance (Å) *versus* time (fs) for trajectories started at the continuum solvent transition state TS1 and moving in the forward direction towards leaving group loss.

With the observation of the intimate ion pair intermediate from the molecular dynamics trajectories we wondered if the intermediate was formed as an inherent intermediate or the result of the TMSCN solvent cage. Therefore, we removed all the solvents from TS1 except the single TMSCN that acts as a nucleophile and re-optimized the structure as a potential energy structure in continuum solvent. This resulted in a fully optimized transition-state structure shown in Fig. 4, which is for carbon-leaving group bond heterolysis. We note that TS2 could not be located as a continuum solvent transition-state structure. With the continuum solvent version of TS1 we performed quasiclassical direct dynamics trajectories. The bottom of Fig. 4 plots the carbon–oxygen bond breaking distance *versus* time for 10 trajectories. This plot shows that a few trajectories show carbon–oxygen distances of >6 Å under 1000 fs and before 2 ps most trajectories have very long distances, which indicates formation of a separated ion pair intermediate. This indicates that the intimate ion pair intermediate found with trajectories from TS1 is the result of a solvent cage dampening the motion of the [(TFA)Sc(OTf)_3_]^−^ leaving group.

**Fig. 5:**
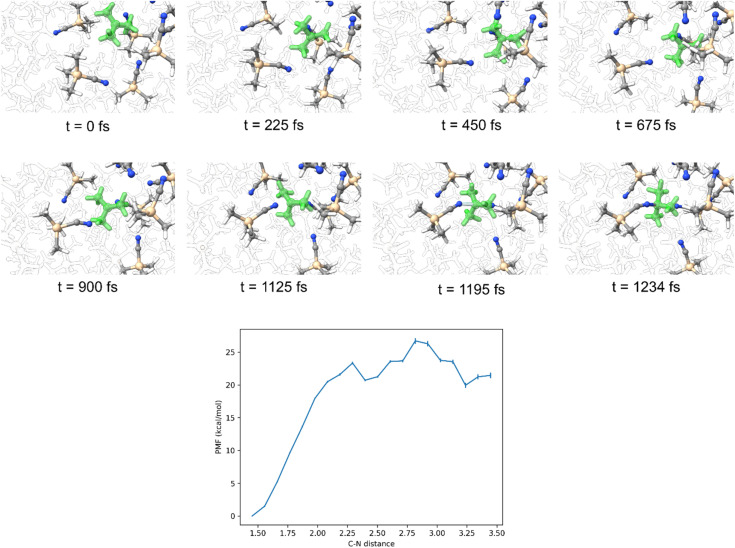
Top: molecular dynamics simulation snapshots of bare *tert*-butyl cation in TMSCN solvent. Several TMSCN molecules that started close to the carbocation were included in the DFT ONIOM layer. The remainder of the solvents were treated with UFF, which are fully opaque. Bottom: PMF curve for C–N distance. This system contains the *tert*-butyl cation, one TMSCN molecule that is in the DFT ONIOM layer, and 150 TMSCN molecules that are in the UFF ONIOM layer.

Having established that the intimate ion pair in this reaction is the result of a solvent cage we also wanted to determine if the lack of dynamically coupled leaving group loss and nucleophilic attack is because of the [(TFA)Sc(OTf)_3_]^−^ leaving group remaining tightly coordinated to the *tert*-butyl cation or if the TMSCN nucleophile is not strong enough to efficiently capture the cation. Therefore, we solvated *tert*-butyl cation with TMSCN, equilibrated the solvent, and then allowed the system to evolve over time without any constraints. Fig. 5 shows that this resulted in trajectories forming the [TMSCN-C(CH_3_)_3_]^+^ adduct within 1.5 ps. We also estimated the barrier for TMSCN capturing *tert*-butyl cation using an umbrella sampling technique with a harmonic potential to generate a potential of mean force (PMF) surface (Fig. 5). Consistent with the relatively fast capture of the *tert*-butyl cation from the direct dynamics trajectories, the barrier for forming the [TMSCN-C(CH_3_)_3_]^+^ is less than 5 kcal mol^−1^. This barrier and the trajectory results indicate that the leaving group prevents immediate *tert*-butyl cation reaction with TMSCN and therefore requires a pathway with TS2 where the leaving group is displaced from the intimate ion pair intermediate through an S_N_2-type transition state.

## Conclusion

Quasiclassical/classical direct dynamics simulations (using M06-2X/UFF ONIOM) were used to find dividing surface transition states for the substitution reaction of nucleophile TMSCN with electrophile [^*t*^Bu-OTFA-Sc(OTf)_3_] in a sphere of explicit TMSCN solvent. The first transition state for bond heterolysis has some engagement of the nucleophile but exclusively leads to an intimate ion pair intermediate. Perhaps surprisingly, trajectories with only implicit solvent show that the leaving group is completely ejected and therefore the explicit solvent cage is responsible for formation of the intimate ion pair. The second transition state for nucleophile attack involves dynamically coupled motion for forming the nitrogen–carbon bond and leaving group complete ejection, which is essentially an S_N_2 type transition state. This perspective was confirmed because dynamics simulations of a bare carbocation showed extremely fast nucleophilic attack.

## Author contributions

A. J. Schaefer developed code to run simulations, executed simulations, analyzed data, and wrote the manuscript. T. Mallavia executed simulations and analyzed data. D. H. Ess designed the simulations, analyzed data, and wrote the manuscript.

## Conflicts of interest

There are no conflicts to declare.

## Supplementary Material

SC-OLF-D5SC05616K-s001

## Data Availability

The data supporting this article have been included as part of the supplementary information (SI). Supplementary information is available. See DOI: https://doi.org/10.1039/d5sc05616k.
