# Supplementary material for: Dynamics-based transition states reveal solvent cage effect and SN2 transition state motion in Lewis acid catalyzed stereoselective tertiary alcohol nucleophilic substitution reactions
Source: Chem Sci. 2025 Oct 16;16(45):21554–61. doi: 10.1039/d5sc05616k (PMC12529080; doi:10.1039/d5sc05616k)
Supplement: SC-016-D5SC05616K-s001 [file SC-016-D5SC05616K-s001.pdf]

Supplementary Information (SI) for Chemical Science.
This journal is © The Royal Society of Chemistry 2025

Electronic Supplementary Information

Dynamics-Based Transition States Reveal Solvent Cage Effect and S_N2 Transition State Motion in Lewis Acid Catalyzed Stereoselective Tertiary Alcohol Nucleophilic Substitution Reactions

Anthony J. Schaefer, Trevor Mallavia, and Daniel H. Ess*

Department of Chemistry and Biochemistry, Brigham Young University, Provo, Utah, 84604, United States

Contents

Molecular Structures and Energies 2
  Breaking C-O Bond Saddle Point 2
  Breaking C-O Bond Dynamic TS 3
  Forming C-N Bond Dynamic TS 5
Trajectory Details 7
  Results of Trajectories Started from Breaking C-O Bond Dynamic TS 7
  Results of Trajectories Started from Forming C-N Bond Dynamics TS 8
  Umbrella Sampling Procedure 8
Alternative Coordination Model 9
Sample Input Files 14
  Packmol Adding TMSCN Solvent 14
  Gaussian Input for Force Computation 15
References 15

Molecular Structures and Energies

Breaking C-O Bond Saddle Point

Imaginary frequency: 130.2264*i* cm^{-1}
Electronic Energy: -4116.04011432 E_h
60 atoms

C	-3.20762	-2.10274	-0.87978
C	-2.46616	-2.08025	-2.15308
H	-1.43234	-2.39994	-2.02460
H	-2.98658	-2.82950	-2.77061
H	-2.53526	-1.11496	-2.65476
C	-4.46417	-1.34015	-0.79455
H	-5.15433	-1.93422	-1.41647
H	-4.87419	-1.29667	0.21263
H	-4.38031	-0.35409	-1.25292
C	-2.97619	-3.22195	0.04815
H	-1.92876	-3.52244	0.06552
H	-3.36099	-3.01828	1.04653
H	-3.56703	-4.04496	-0.38542
C	-1.83842	0.17131	0.83675
C	-3.05928	0.37287	1.75583
F	-2.74531	1.05691	2.84920
F	-4.01196	1.04153	1.09778
F	-3.56231	-0.80794	2.12651
O	-0.91640	1.03382	0.99777
Sc	1.06731	1.06998	0.56486
O	1.15096	-0.42702	-1.00758
O	3.05946	1.24862	0.69205
O	0.80997	2.30500	-1.18522
S	4.31746	1.37322	1.54665
S	1.19937	-1.62936	-0.10210
S	0.77596	3.62998	-0.46542
O	4.47029	0.25059	2.45198
O	5.44925	1.81774	0.75853
O	0.94467	3.24364	0.97363
O	-0.28020	4.54873	-0.80433
O	0.35662	-2.75799	-0.41007
O	1.10979	-1.02673	1.27048
C	2.92164	-2.26951	-0.23614
C	3.82785	2.79447	2.59807

C	2.35489	4.45476	-0.92877
F	3.79131	-1.27717	-0.18604
F	3.03957	-2.90911	-1.38731
F	3.13096	-3.10429	0.76784
F	4.76450	3.01339	3.50992
F	2.67931	2.51359	3.21094
F	3.66969	3.88357	1.85865
F	2.25511	4.88460	-2.17503
F	3.35776	3.60188	-0.82785
F	2.54643	5.47703	-0.11188
O	-1.83311	-0.74839	0.02293
Si	-7.38639	-6.09850	-3.30392
C	-6.64457	-7.80932	-3.34076
H	-7.39961	-8.52980	-3.67210
H	-5.79981	-7.85912	-4.03381
H	-6.29833	-8.11182	-2.34828
C	-7.87831	-5.47134	-4.99016
H	-8.69235	-6.08687	-5.38705
H	-8.22807	-4.43615	-4.94055
H	-7.04044	-5.52196	-5.69148
C	-8.73136	-5.90608	-2.02604
H	-9.08031	-4.87089	-1.97093
H	-9.58421	-6.53869	-2.29352
H	-8.38173	-6.20842	-1.03468
C	-5.97952	-4.95237	-2.73057
N	-5.12340	-4.25429	-2.38172

Breaking C-O Bond Dynamic TS

C	-1.04408	-4.81730	6.83963
C	-0.42581	-4.46342	5.53674
H	0.65265	-4.61164	5.56363
H	-0.85161	-5.13515	4.78719
H	-0.67556	-3.43859	5.25560
C	-2.32455	-4.15782	7.21014
H	-2.96866	-4.11976	6.33010
H	-2.82787	-4.66337	8.03374
H	-2.12103	-3.11938	7.48160
C	-0.48965	-5.92201	7.66195
H	0.57352	-6.05889	7.46789
H	-0.67466	-5.73318	8.71986
H	-1.02403	-6.83568	7.39193

C	0.45109	-2.66913	8.66915
C	-0.61957	-2.59863	9.77649
F	-0.09216	-2.24351	10.94271
F	-1.54394	-1.69067	9.43849
F	-1.23113	-3.77416	9.92899
O	1.38557	-1.81353	8.81896
Sc	3.24355	-1.54431	8.07140
O	3.10426	-2.78946	6.29589
O	5.21115	-1.22691	7.82996
O	2.61060	-0.07604	6.62239
S	6.59965	-1.17171	8.45793
S	3.45434	-4.08856	6.97107
S	2.65973	1.11942	7.54097
O	6.95458	-2.42244	9.10135
O	7.55300	-0.52282	7.58038
O	3.11789	0.53671	8.84404
O	1.51915	1.99939	7.55213
O	2.66046	-5.25399	6.66924
O	3.61258	-3.69566	8.41117
C	5.15262	-4.50704	6.38596
C	6.25538	0.00807	9.81902
C	4.08243	2.12033	6.93947
F	5.91463	-3.43002	6.34916
F	5.05542	-5.02450	5.17306
F	5.66624	-5.39693	7.21787
F	7.32491	0.12573	10.59258
F	5.23905	-0.45347	10.54715
F	5.93267	1.19687	9.32839
F	3.72738	2.72364	5.81764
F	5.12629	1.34295	6.71925
F	4.37656	3.01976	7.86361
O	0.33034	-3.46262	7.74266
Si	-4.34937	-8.52763	4.33848
C	-3.38915	-10.12598	4.37515
H	-3.97858	-10.91993	3.90489
H	-2.44653	-10.03288	3.82821
H	-3.16669	-10.43011	5.40198
C	-4.63846	-7.87771	2.61477
H	-5.28770	-8.56856	2.06679
H	-5.12558	-6.89867	2.63730
H	-3.69786	-7.78582	2.06406

C	-5.89244	-8.56659	5.38474
H	-6.38070	-7.58806	5.40630
H	-6.59984	-9.29112	4.96805
H	-5.66569	-8.86503	6.41230
C	-3.21227	-7.24695	5.17376
N	-2.51808	-6.47230	5.68441

Forming C-N Bond Dynamic TS

C	-1.11275	-5.62407	7.40579
C	0.20781	-5.49394	6.75738
H	0.59625	-4.53707	7.14291
H	0.89803	-6.27693	7.07018
H	0.12795	-5.42736	5.67243
C	-2.25258	-4.88613	6.83046
H	-2.23561	-4.88139	5.74080
H	-3.20979	-5.22679	7.22417
H	-2.08785	-3.85244	7.17651
C	-1.16581	-6.00637	8.82863
H	-0.43410	-6.77386	9.07751
H	-0.86547	-5.08297	9.35093
H	-2.17244	-6.27789	9.14567
C	0.60035	-1.72673	8.61070
C	-0.47372	-0.76355	8.07508
F	-0.69261	0.22399	8.94933
F	-0.07236	-0.21186	6.92521
F	-1.62949	-1.38940	7.85600
O	1.73274	-1.13053	8.77936
Sc	3.59716	-1.78886	9.08119
O	3.23249	-3.73708	8.17034
O	5.53245	-2.17109	9.54194
O	4.12530	-1.32658	7.02287
S	6.61206	-2.20216	10.61168
S	2.90922	-4.45949	9.45240
S	4.45017	0.12499	7.25420
O	6.19147	-2.92837	11.79616
O	7.92116	-2.46453	10.04555
O	4.25485	0.30449	8.73002
O	3.84979	1.09402	6.37160
O	1.79273	-5.37014	9.47641
O	2.97283	-3.37227	10.48504
C	4.37899	-5.52528	9.78785

C	6.59956	-0.43081	11.08387
C	6.26781	0.25469	6.98921
F	5.49999	-4.90941	9.45656
F	4.25865	-6.63304	9.07330
F	4.39134	-5.82204	11.07650
F	7.41630	-0.23076	12.10915
F	5.36327	-0.08137	11.43767
F	6.98399	0.32283	10.06227
F	6.50151	0.24830	5.68668
F	6.89339	-0.75826	7.55843
F	6.68485	1.39555	7.51480
O	0.33310	-2.89114	8.82022
Si	-2.02100	-10.42222	5.42660
C	-0.83818	-11.50916	6.37011
H	-0.92818	-12.54076	6.01420
H	0.19710	-11.18793	6.22474
H	-1.06044	-11.49774	7.44095
C	-1.61523	-10.25656	3.61590
H	-1.75250	-11.22367	3.12125
H	-2.27106	-9.52920	3.12912
H	-0.57744	-9.94370	3.47007
C	-3.81520	-10.78973	5.76797
H	-4.46929	-10.06633	5.27303
H	-4.06104	-11.78710	5.38885
H	-4.02447	-10.77280	6.84135
C	-1.74095	-8.66822	6.14430
N	-1.57040	-7.60927	6.57545

Trajectory Details

Results of Trajectories Started from Breaking C-O Bond Dynamic TS

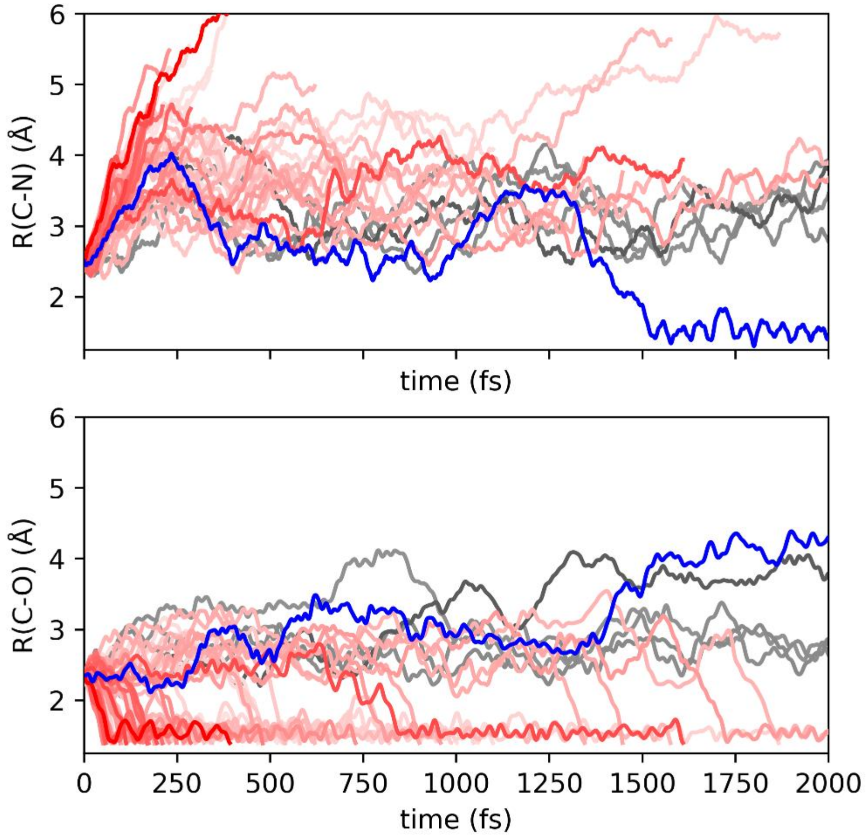

Figure S1: Plot of C-N (top) and C-O (bottom) distances for trajectories starting from dynamics TS1. Red indicates the 55 trajectories that form a C-O bond, blue indicates the one trajectory that formed the C-N bond, and blue indicates the 4 trajectories that were still at the carbocation intermediate at the end of the trajectory.

Results of Trajectories Started from Forming C-N Bond Dynamics TS

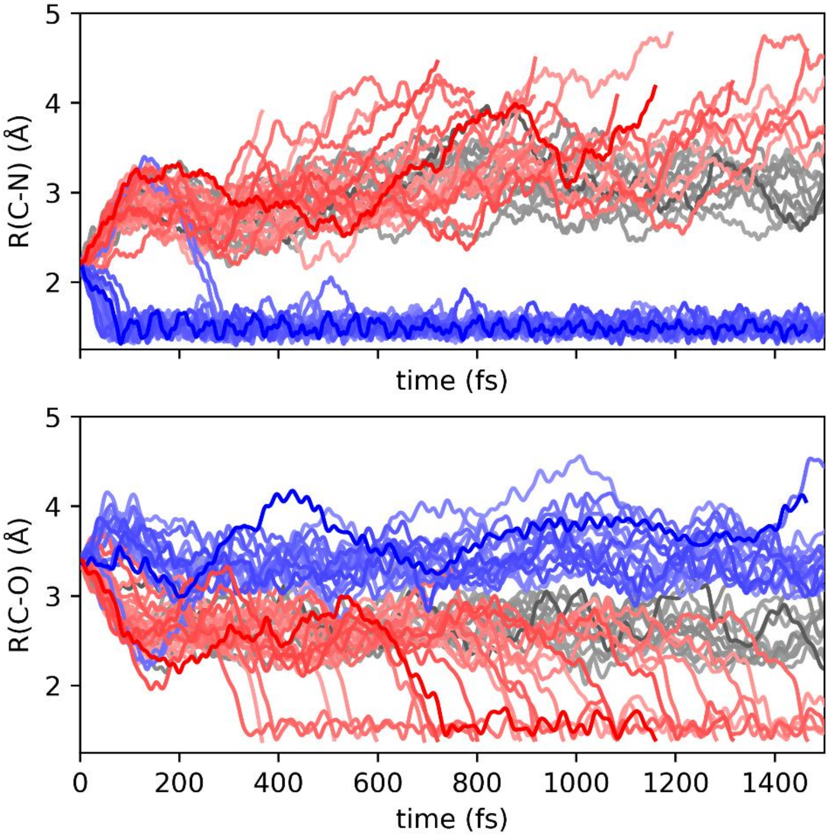

Figure S2: Plot of C-N (top) and C-O (bottom) distances for trajectories starting from dynamics TS2. Red indicates the 22 trajectories that form a C-O bond, blue indicates the 17 trajectories that form the C-N bond, and blue indicates the 11 trajectories that were still at the carbocation intermediate at the end of the trajectory.

Umbrella Sampling Procedure

Umbrella sampling was carried out with our Milo 1.2 software interfaced to Gaussian. A harmonic force constant of 825 kcal/mol•Å^2 was used to restrain the C-N bond length to different regions of the reaction coordinate. Each window was equilibrated for at least 12.5 ps, and production runs sampled for at least 7.5 ps. A CSVR thermostat was used to keep the system at a constant 298.15 K throughout both equilibration and production runs. The potential of mean force curve was then calculated using the WHAM software.[1]

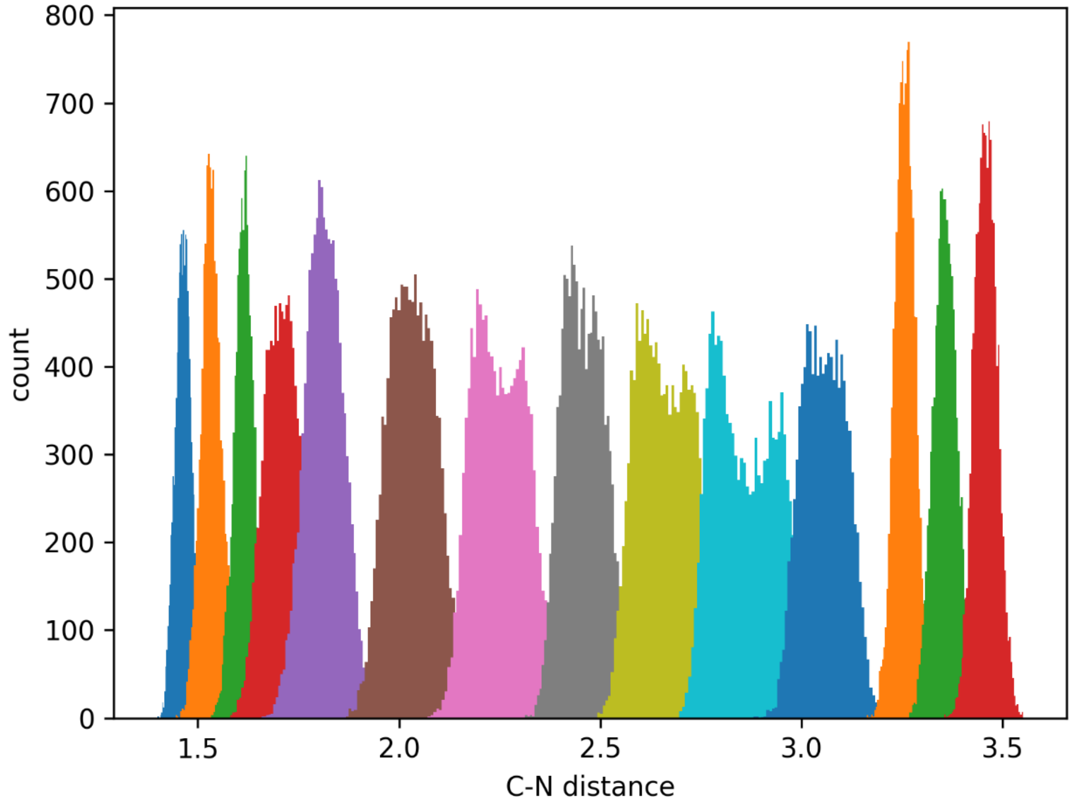

Figure S3: plot of distribution of sampling along the C-N distance for individual windows during production runs for umbrella sampling.

Alternative Coordination Model

We have also considered the possibility of TMSCN solvent molecules replacing triflates in coordination to the scandium center. We located the structure below using the M06-2X/6-31G(d,p) with LANL2DZ for Sc and PCM(acetonitrile). Ten classically sampled and ten quasiclassically sampled trajectories were from a structure analogous to dynamic TS1 in implicit solvent. None of these trajectories formed the C-N bond within the first 500 fs. Two classically sampled trajectories reformed the C-O bond. Several trajectories ended early due to related to the PCM algorithm.

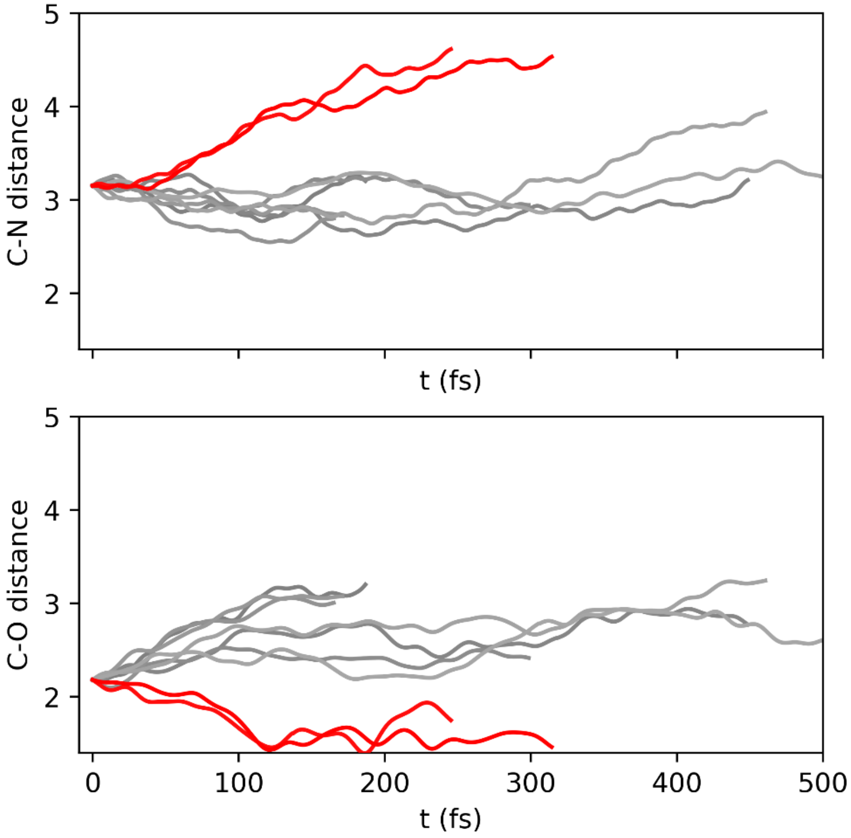

Figure SI4: Plot of C-N (top) and C-O (bottom) distances for classically sampled trajectories starting from the structure below

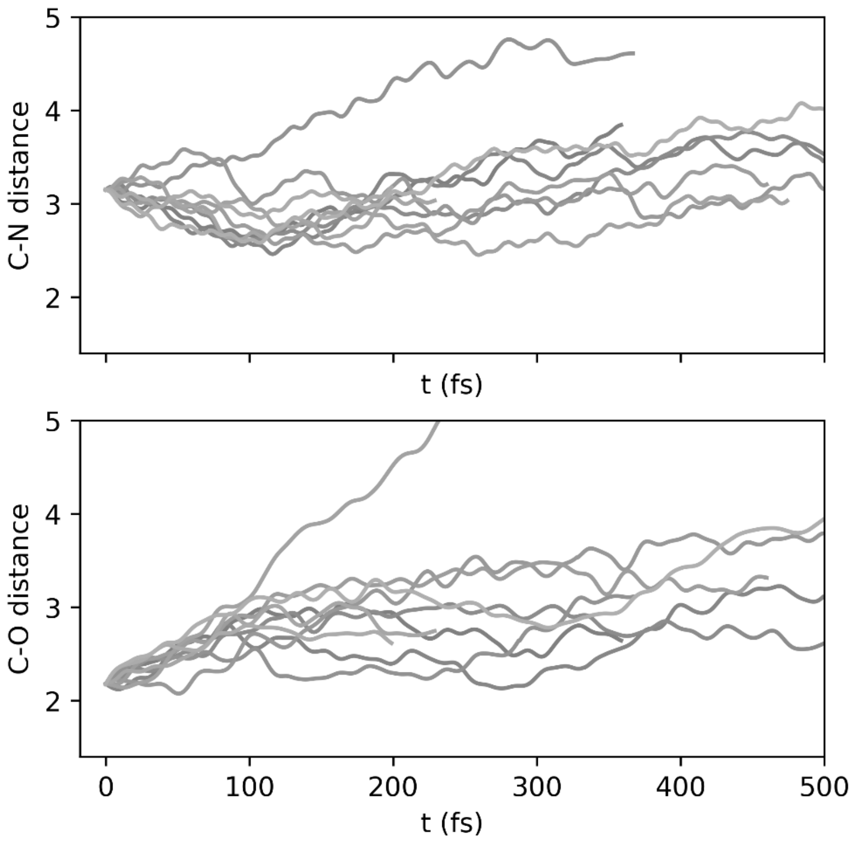

Figure S5: Plot of C-N (top) and C-O (bottom) distances for quasiclassically sampled trajectories starting from the structure below

Approximate dynamic TS1 with five TMSCN coordinated to Sc

Sc	1.52227	0.12558	-0.17466
N	2.74772	-0.37683	1.61038
C	4.73623	-3.15445	3.58563
H	5.25233	-3.64117	4.41827
H	3.85808	-3.75301	3.33083
H	5.41299	-3.13598	2.72760
C	2.95076	-1.36025	5.43341
H	3.37849	-1.73137	6.36902
H	2.60913	-0.33519	5.59714
H	2.08987	-1.98376	5.18098
C	5.67498	-0.25954	4.33738
H	5.32183	0.75965	4.51125
H	6.25348	-0.57497	5.21053

H	6.33836	-0.26116	3.46939
Si	4.24771	-1.43134	4.09745
C	3.33154	-0.74399	2.53940
N	0.31012	-1.72010	0.33180
C	0.39760	-5.24305	2.05827
H	0.04200	-6.20297	2.44365
H	1.18852	-5.43932	1.33070
H	0.81942	-4.67594	2.89204
C	-1.71059	-5.13563	-0.25824
H	-2.20255	-6.07205	0.02019
H	-2.44874	-4.49813	-0.74995
H	-0.91451	-5.36614	-0.97016
C	-2.32558	-3.77804	2.49770
H	-3.09302	-3.16477	2.01862
H	-2.81217	-4.66046	2.92348
H	-1.87742	-3.20808	3.31562
Si	-1.02923	-4.32858	1.27743
C	-0.21614	-2.69466	0.66300
N	2.54275	-1.66177	-1.11379
C	2.51637	-5.37939	-2.47719
H	2.91226	-6.33208	-2.84005
H	1.95808	-5.57078	-1.55744
H	1.83069	-4.98436	-3.23066
C	5.02903	-4.70741	-0.74491
H	5.54785	-5.63563	-1.00121
H	5.77991	-3.94133	-0.53681
H	4.44348	-4.88375	0.16123
C	4.84473	-3.67459	-3.69948
H	5.59292	-2.91317	-3.46769
H	5.35889	-4.53958	-4.12828
H	4.15815	-3.27869	-4.45179
Si	3.93074	-4.20598	-2.16615
C	3.07239	-2.60201	-1.52871
N	3.31060	0.88673	-1.33313
C	6.75949	2.82867	-1.77785
H	7.63042	3.23678	-2.29860
H	6.24168	3.65376	-1.28310
H	7.11279	2.12604	-1.01947
C	4.82556	3.15585	-4.20976
H	5.58765	3.60199	-4.85520
H	4.10252	2.63586	-4.84232

H	4.31484	3.96183	-3.67680
C	6.39886	0.47313	-3.80331
H	5.66646	-0.06293	-4.41135
H	7.22468	0.77861	-4.45225
H	6.79516	-0.20600	-3.04430
Si	5.64973	1.98686	-3.01585
C	4.19158	1.29775	-1.95920
N	1.81884	2.30819	0.34453
C	2.43634	5.36120	2.82876
H	2.59690	6.39914	3.13464
H	1.54293	4.99013	3.33637
H	3.29788	4.77341	3.15459
C	0.70210	6.12167	0.33063
H	0.77332	7.19924	0.50497
H	0.58785	5.95696	-0.74344
H	-0.18939	5.75137	0.84264
C	3.79516	5.71938	0.03510
H	3.68434	5.49280	-1.02862
H	4.00391	6.78789	0.13822
H	4.65120	5.16507	0.42803
Si	2.24382	5.29633	0.97613
C	1.97136	3.42788	0.58910
N	-0.06945	0.72051	1.33146
C	-2.15998	3.40375	3.36406
H	-2.75377	3.82055	4.18267
H	-1.22343	3.96345	3.30226
H	-2.71583	3.54280	2.43343
C	-0.71398	1.28521	5.15398
H	-1.22333	1.59160	6.07230
H	-0.46587	0.22429	5.23942
H	0.21265	1.85834	5.06876
C	-3.35805	0.52526	3.66650
H	-3.09586	-0.53581	3.66479
H	-3.95372	0.72697	4.56148
H	-3.98125	0.73974	2.79480
Si	-1.83525	1.59985	3.69780
C	-0.77028	1.02957	2.19771
O	0.33508	0.50004	-1.73302
O	-1.81727	0.06047	-1.32113
C	-3.99746	0.05987	-1.24144
C	-1.23642	0.90755	-3.47226

```
F     -2.22445    1.81135   -3.44297
F     -0.19471    1.44209   -4.09258
F     -1.66401   -0.14374   -4.17914
C     -0.90474    0.45212   -2.03622
N     -7.11841    0.03914   -0.79767
C     -8.27438    0.06433   -0.72668
Si   -10.17118    0.11110   -0.57935
C    -10.54048   -0.23672    1.21753
H    -11.62335   -0.22533    1.37312
H    -10.09536    0.52155    1.86624
H    -10.16346   -1.21802    1.51581
C    -10.79417   -1.22287   -1.72700
H    -10.48798   -1.03092   -2.75824
H    -11.88737   -1.24769   -1.69445
H    -10.42148   -2.20559   -1.42834
C    -10.67199    1.82937   -1.11001
H    -10.37206    2.02636   -2.14208
H    -10.22294    2.58796   -0.46425
H    -11.75968    1.92666   -1.04562
C     -4.34751   -0.27448   -2.63318
H     -3.75422   -1.11024   -3.00341
H     -4.29488    0.57933   -3.30756
H     -5.39553   -0.60331   -2.56530
C     -4.10789    1.45486   -0.78652
H     -3.73093    2.14591   -1.53939
H     -3.64496    1.62069    0.18482
H     -5.19517    1.60224   -0.69149
C     -3.97000   -1.02342   -0.24398
H     -3.69500   -1.98365   -0.67873
H     -5.01460   -1.08072    0.10240
H     -3.33657   -0.77118    0.60665
```

Sample Input Files

Packmol Adding TMSCN Solvent

```
tolerance 2.5
filetype xyz
output packmol_structure.xyz

structure packmol_solute.xyz
```

```
  number 1
  fixed 0. 0. 0. 0. 0. 0.
end structure

structure packmol_TMSCN.xyz
  number 150
  inside sphere 0.0 0.0 0.0 20.0
end structure
```

Gaussian Input for Force Computation

```
#n oniom(M062X/genecp:UFF=print) force geom=(connectivity)

title

0 1 0 1 0 1
<molecular structure, including connectivity info>

C F H N O S Si 0
6-31G(d,p)
****
Sc 0
lanl2dz
****

Sc 0
lanl2dz
```

References

1. Bauer, D., *WHAM - An efficient weighted histogram analysis implementation written in Rust*, Ver 1.1.3. https://doi.org/10.5281/zenodo.1488597
